# Supplementary material for: Rapid detection of isthmus block and rhythm change using local electrogram changes during complex atrial flutter ablation
Source: Europace. 2022 Sep 15;25(2):756–61. doi: 10.1093/europace/euac161 (PMC10103578; doi:10.1093/europace/euac161)
Supplement: euac161_Supplementary_Data [file euac161_supplementary_data.zip › ComplexFlutterTrackedChanges.docx]

**Rapid Detection of Isthmus Block and Rhythm Change Using Local Electrogram Changes During Complex Atrial Flutter Ablation**

Benzy J. Padanilam MD^1^, Sarah W. Whittam, MS^2^, Brad A. Clark DO^1^, Jeffrey A. Olson DO^1^, Girish V. Nair MD^1^, Sandeep A. Joshi MD,^1^ Eric N. Prystowsky MD^1^ , Parin J. Patel MD^1^, Jasen L. Gilge MD^1^

Address for Correspondence:

Benzy J. Padanilam, MD

8333 Naab Road, #400

Indianapolis, IN 46260

Phone: 3173386100

e-mail: [bjpadani@ascension.org](mailto:bjpadani@ascension.org)

^1^Ascension St Vincent**,** 8333 Naab Road, #400**,** Indianapolis, IN 46260. Division of Cardiology

^2^Abbott Laboratories, 100 Abbott Park Road, Abbott Park, IL 60064

Conflicts of interest or financial disclosures: SWW is an employee of Abbott Laboratories. No other authors have conflict of interest.

**Rapid Detection of Isthmus Block and Rhythm Change Using Local Electrogram Changes During Complex Atrial Flutter Ablation**

Multiple reentry circuits may operate simultaneously in the atria in the form of dual loop reentry using a common isthmus, or multiple reentrant loops without a common isthmus. When two or more reentrant circuits coexist, ablation of an individual isthmus may lead to a seamless transition (without significant changes in surface ECG, coronary sinus activation or tachycardia cycle length) to a second rhythm, and the isthmus block can go unnoticed. It is important to recognize an isthmus block promptly to avoid prolonged unnecessary ablations at an already blocked site. Electroanatomic remapping of the entire circuit to identify rhythm changes is time consuming and difficult to do frequently. Repeat entrainment mapping could be challenging due to difficulty with capture thresholds and decremental conduction in areas of scar. We describe methods to rapidly identify a transition in the rhythm using local electrogram changes at the ablation site.

**Hypothesis:**

In a reentrant circuit, the local electrograms at an isthmus may be considered in terms of the depolarization wavefront approaching the site (approaching wavefront, Awf) and the wavefront receding from the site (receding wavefront, Rwf) (Figure 1). When two simultaneous circuits are operative, the rhythm can be a figure of eight (dual loop) reentry (1) involving a common isthmus (Figure 1) or two independent reentrant circuits with separate isthmuses (Figure 2). Even in a dual loop reentry there may be additional independent isthmuses that are unique only to one of the reentrant loops (non-common isthmus). Ablation of the common isthmus will result in termination of a dual loop reentry while ablation of the non-common isthmus would result in local block and transition of the rhythm to the second reentry (2). We hypothesized that when conduction block occurs at a non-common isthmus, the reentry circuit would change with alteration of local activation at the site. The following would indicate rhythm change and isthmus block:

1. *Activation sequence:* Reversal of activation sequence at the Rwf side, with or without reversal of activation at the Awf side of ablation line.

2. *Single electrogram timing:* advancement of electrogram timing on the Rwf side of ablation line; advancement or delay of electrogram timing at the Awf side.

3. *Double potentials (DPs):* Merging of preexisting DPs.

Any of these changes would indicate that the direction of activation at the isthmus being ablated has changed. Change in the direction of activation implies a change in the rhythm, most likely a result of isthmus block at the site of ablation. In the case of a dual loop reentry, block at the non-common isthmus will lead to reversal of activation only at the Rwf (Figure 1). In the case of two rhythms without a common isthmus, ablation of an individual isthmus could lead to change of activation on the Rwf and Awf (Figure 2). The exact changes would depend on the location and direction of activation from the second reentry, and the nature and location of the isthmus. Finally, reentry around a defined scar typically features wide double potentials (DP) due to temporal separation of activation wavefronts on either side of the line of block. Merging of these DPs during ablation denotes change of activation at the site and isthmus block. Three representative cases presented below demonstrate these principles. Supplemental files are provided including video propagation maps and complete lesion sets of the three cases, and procedural details of case 1.

Figure 1. Dual Loop Reentry. Panel A shows a model for dual loop reentry with a common isthmus (CI) and a non-common isthmus (NCI). The activation wavefront approaching the isthmus is called the approaching wavefront (Awf) and the wavefront moving away from the isthmus is called the receding wavefront (Rwf). Note the activation reversal at the Rwf area after block in the NCI in Panel B.

Figure 2. Multiloop reentry without common isthmus. Panel A shows a model for two reentrant circuits with no common isthmus. Each circuit has an individual non-common isthmus (NCI). The approaching wavefront (Awf) and the receding wavefront (Rwf) are as defined in Figure 1. In Panel B, note that the activation changes at the Rwf and Awf areas with block in the NCI of one of the circuits. A narrow and confined isthmus is depicted, and the wavefront must enter it at the edges leading to a reversal of Rwf area activation. If the isthmus is broad, it could be activated without reversal at the Rwf or Awf, but relative timing would still change.

*Case 1: Dual Loop Reentry (Mitral + Roof flutter)*

A 72-year-old man presented with recurrent atypical flutter after previous pulmonary vein isolation and typical cavo-tricuspid isthmus (CTI) dependent flutter ablations. Electroanatomic mapping of the presenting rhythm showed dual loop reentry with counterclockwise mitral reentry and roof dependent reentry around the left pulmonary veins (Figure 3A). Ablation lesions between left and right superior pulmonary veins posteriorly (roof line) led to a 10ms slowing of the TCL (230 to 240ms). Surface ECG and coronary sinus activations remained unchanged (Figure 4). It was unclear if the roof line blocked or whether the small increase in TCL was due to conduction delay. The activation sequence at the Rwf was continuously being evaluated during ablation with a multipolar catheter (HD grid^TM^, Abbott Laboratories) and a reversal of the activation wavefront was noted coinciding with the TCL change (Figure 3C). This reflected a change in rhythm and block across the roof line. The mitral activation was delayed by 10ms because the roof reentry was the faster loop in this case. However, the reversal of activation at the Rwf would have been indicative of rhythm change and isthmus block even without TCL change. Further electroanatomic mapping confirmed a counterclockwise mitral flutter with no further roof reentry (Figure 3B). A mitral isthmus ablation between the left inferior pulmonary vein and mitral valve terminated the tachycardia and differential pacing at the roof and mitral lines indicated block at both sites.

Figure 3. Dual Loop Reentry

Panel A shows dual loop reentry around the mitral valve and left pulmonary veins. Lateral mitral isthmus is common to both loops, and the roof is a non-common isthmus involving only the reentry around the left pulmonary veins. Panel B shows continued counterclockwise mitral reentry after roof block from ablation lesions represented as yellow spheres. Note the reversal of activation occurring only at the Rwf of the ablation site, and the remaining left atrial activation remains the same. A mitral isthmus ablation between left inferior pulmonary vein and mitral annulus terminated the tachycardia. Panel C shows electrogram sequence reversal with unchanged surface ECG and coronary sinus activations at the time of rhythm change. The bottom six electrograms are from A and B splines of the HD Grid^TM^ mapping catheter located at the Rwf. The arrows show reversal of activation sequence. Tachycardia cycle length (TCL) changes from 230ms to 240ms. The color scheme of the isochrones is shown at the top with white representing the earliest and purple representing the latest electrograms. LAA- left atrial appendage, LPV- left pulmonary veins, MV- mitral valve, RPV- right pulmonary veins, Awf- approaching wavefront, Rwf- receding wavefront.

Figure 4. Seamless rhythm transition. The tachycardia cycle length increases from 230ms to 240ms but no appreciable changes in the surface ECG or coronary sinus activation are noted.

*Case 2: Two reentrant circuits without a common isthmus*

A 52-year-old man with previous history of ASD repair presented with ECG morphology suggestive of typical CTI flutter. Electroanatomic mapping revealed reentry around an atriotomy scar anterior to the SVC (Figure 5A). The TCL was 285ms, and wide DPs were apparent with blue isochrones adjacent to white isochrones at the mid part of the scar. No simultaneous reentry was noted around the tricuspid valve, but a potential lower loop clockwise reentry around the inferior vena cava was noted. An ablation line was performed from TV to the atriotomy scar. No tachycardia termination, change in TCL, change in CS activation, or change in surface ECG occurred despite anatomic completion of the ablation line. However, the wide DPs seen previously at the atriotomy site were no longer seen and replaced by closely spaced electrograms (Figure 5C). Conversion of DPs to closely spaced electrogram was interpreted as indicative of a rhythm change and block of the isthmus being ablated. Additionally, the Rwf electrogram advanced (blue to red isochrone) and Awf electrogram advanced (blue to orange isochrone), both indicating a rhythm change. Remapping of the rhythm was undertaken revealing no further scar reentry but continued lower loop reentry around the inferior vena cava (Figure 5B). A CTI ablation terminated the lower loop reentry.

Figure 5. Scar reentry and lower loop reentry

Panel A shows the presenting reentry around right atrial scar just below superior vena cava. Wide double potentials (DP) are apparent at the mid-scar (dotted black line) indicated by white isochrones adjacent to blue isochrones. Electrograms at the scar from the area marked by the star is shown on the right with dotted arrows pointing to early electrogram from red isochrone and later electrogram from the light blue isochrone forming the DP. The ablation catheter can be seen pointing to the area of planned ablation line from the tricuspid valve to the scar; dotted arrow is shown pointing to the electrogram at the site. Simultaneous reentry around the inferior vena cava (lower loop reentry) is also seen in the view through the tricuspid valve. Collision of activation wavefronts rules out reentry around the tricuspid valve. Panel B shows remapping of the rhythm after ablation line (represented as red spheres) completion from tricuspid valve to the scar. Continuation of lower loop reentry around the inferior vena cava with passive activation around the atriotomy scar is now apparent. Simultaneous activation of anterior and posterior aspect of the scar results in the loss of double potentials (noted as white and red isochrones) along the scar. Dotted arrow pointing to electrogram at the scar from the same location marked by the star from Panel A is shown with merging of the previous DP. Also note that the Rwf and Awf electrograms advance; Rwf electrogram is shown from the ablation catheter tip with dotted arrow- the electrogram was in the blue isochrone in Panel A and is in the red isochrone in Panel B. Any of these changes is indicative of rhythm change and isthmus block. Panel C shows the surface and intracardiac electrograms during rhythm change, with ablation catheter at the starred position in Panels A/B. The TCL (285 ms), surface ECG and CS electrograms remain unchanged while the ablation electrograms recorded at the scar change from wide double potentials to closely spaced early electrograms (arrows). The color scheme of the isochrones is shown at the top with white representing the earliest and purple representing the latest electrograms. IVC- inferior vena cava, SVC- superior vena cava, TV- tricuspid valve, CS- coronary sinus

***Case 3***

A 74-year-old man presented for ablation of typical CTI flutter and 3D electroanatomic map demonstrated dual loop reentry with counterclockwise typical flutter and intra isthmus reentry around a scar mid-way in the CTI. The common isthmus of the dual loop was the part of the CTI posterior to the scar and the non-common isthmus was the part of the CTI anterior to the scar. Ablation was started at the TV end of the CTI, and wide DP formation was noted during ablation of the CTI anterior to the scar (Figure 6) without change in TCL, ECG morphology or CS activation. The timing of the first component of the DP was the same as previous electrograms at the site while the second component was delayed either from block or conduction delay at the site. Further electroanatomic mapping was not undertaken and completion of the CTI ablation posterior to the scar (common isthmus) terminated the tachycardia. Bidirectional CTI block was confirmed.

Figure 6: Dual loop (Intraisthmus and tricuspid valve) reentry. Electroanatomic map in Panel A shows intra isthmus reentry in the cavotricuspid isthmus (CTI) along with typical counterclockwise reentry around tricuspid valve (TV). Blue dotted arrows point to electrogram change at the site of ablation anterior to the mid CTI scar. Panel B shows surface ECG, coronary sinus electrograms and and ablation tip elctrograms at the same site as in panel A. Arrows point to ablation electrogram changing from a single to a double potential (DP) during ablation. The surface ECG is unchanged and the tachycardia cycle length remains unchanged at 210ms. IVC- inferior vena cava, CS- coronary sinus.

**Discussion**

In this report, we characterize methods for quick identification of rhythm change and isthmus block during ablation of complex atrial flutters. We propose that conduction block at a non-common isthmus during multiloop reentry would result in identifiable activation changes at the site. Figure 1 shows the expected changes when a non-common isthmus of a dual loop reentry is ablated. These changes are demonstrated in patient case 1 where mitral and roof flutters form a dual loop reentry. With ablation of the roof (non-common isthmus) the Rwf activation is reversed from the initial pattern, but the rest of the left atrial activation remained unchanged from the mitral reentry. A reversal of activation can be identified with either a multipolar mapping catheter instantaneously (as we did in case 1) or using the roving ablation tip to map a limited area at the Rwf. When a stable multipolar mapping catheter position at the Rwf area is difficult, the catheter may be moved to the area as needed to observe electrogram sequence and timing. The ablation tip electrograms provide a continuous guide for electrogram timing changes at Rwf or Awf areas. If high density mapping of the initial rhythm has been undertaken, one can predict the area where Rwf activation reversal would occur from the line of collision of activation wavefronts during the original dual loop reentry (2). In case 1, one could predict that the Rwf electrogram timing will change to immediately past the reference (light blue on isochronal map) from before the reference (red on isochronal map) when block occurs. Here, one can watch for the Rwf electrograms delaying to the blue isochrone during ablation as likely indicative of block. The timing of the Awf remains unchanged in case 1 despite the increase in TCL because the CS (reference electrode) activation delays equally and the relative timing of electrograms are unaffected.

In the case of two reentrant circuits without a common isthmus, block in one isthmus transitions the rhythm to the second reentry (Figure 2). The exact changes vary depending on the relative locations of the circuits and isthmuses. Reversal of activation of the Rwf is expected if the isthmus is anatomically confined because this area will now be activated from a different wavefront in the opposite direction. If the area is not confined, reversal of activation may not occur, but the timing may advance or delay (Figure 2B). In addition to the Rwf activation changes, the Awf activation could also change here. These changes are demonstrated in patient case 2. Any change (advancement or delay) of the Awf electrogram would indicate rhythm change because ablation of the isthmus should not affect the activation of areas temporally preceding it unless the area is activated differently. It is not necessary to demonstrate activation reversal when changes occur in the Awf- one can use a single electrogram and advancement or delay would be sufficient to show rhythm change. Similarly, any advancement of Rwf electrograms will also indicate a rhythm change because ablation induced slowing of conduction can only delay the Rwf electrograms. The scar reentry in case 2 also illustrates the usefulness of observing DP timing. Merging of the DP electrogram along the scar indicates termination of the reentry around the scar and a rhythm change. A comparison with case 3 illustrates the difference between DP merging versus DP formation during ablation. The former, when confirmed along the scar, indicates rhythm change while the latter can be rhythm change or conduction slowing. It should be noted that rhythm change during ablation of an isthmus could also occur with revelation of a reentrant circuit or a focal tachycardia that was not apparent before. The activation changes we describe here are applicable to occurrence of such unexpected new rhythms.

While the local electrogram changes we describe here should prompt the electrophysiologist to reevaluate the need for continued ablation at the site, it should be used as a guide rather than definitive evidence of isthmus block. Achievement of block does not mean that the ablation at the site should be immediately discontinued. There may be value for further ablation and completion of the anatomic line depending on the stage of ablation. While the changes at the Rwf perhaps provides the most information during an ablation, reversal of Rwf activation may not always indicate block at an isthmus. Significant delay with Rwf reversal can occur due to focal breakthrough conduction on the ablation line or epicardial conduction with breakthrough distant from the line of ablation. Ultimately, Rwf reversal should be taken as likely to indicate block and the state of isthmus conduction further confirmed after rhythm termination.

**Conclusion:**

Local activation sequence changes, electrogram timing and the behavior of pre-existing DPs can detect isthmus block when rhythm transitions occur during ablation of multiloop reentry tachycardias. Expeditious identification of isthmus block, that otherwise may have gone unnoticed, could improve patient safety and outcomes.

**Acknowledgments**

None

**Conflict of interest**

SWW is an employee of Abbott Laboratories. No other authors have conflict of interest.

**References:**

1. Shah D, Jaïs P, Takahashi A, Hocini M, Peng JT, Clementy J, et al. Dual-loop intra-atrial reentry in humans. Circulation. 2000 Feb 15;101(6):631-9. doi: 10.1161/01.cir.101.6.631. PMID: 10673255.
2. Takigawa M, Derval N, Martin CA, Vlachos K, Denis A, Kitamura T, et al. A simple mechanism underlying the behavior of reentrant atrial tachycardia during ablation. Heart Rhythm. 2019 Apr;16(4):553-561. doi: 10.1016/j.hrthm.2018.10.031. PMID: 30389441.

**Supplemental Files**

Supplemental files are provided including video propagation maps and complete lesion sets of the three cases, and procedural details of case 1.
