# Supplementary material for: Rapid detection of isthmus block and rhythm change using local electrogram changes during complex atrial flutter ablation
Source: Europace. 2022 Sep 15;25(2):756–61. doi: 10.1093/europace/euac161 (PMC10103578; doi:10.1093/europace/euac161)
Supplement: euac161_Supplementary_Data [file euac161_supplementary_data.zip › supplementalFigs.pdf]

# Sites of termination of flutter and lesion sets in cases 1, 2 and 3

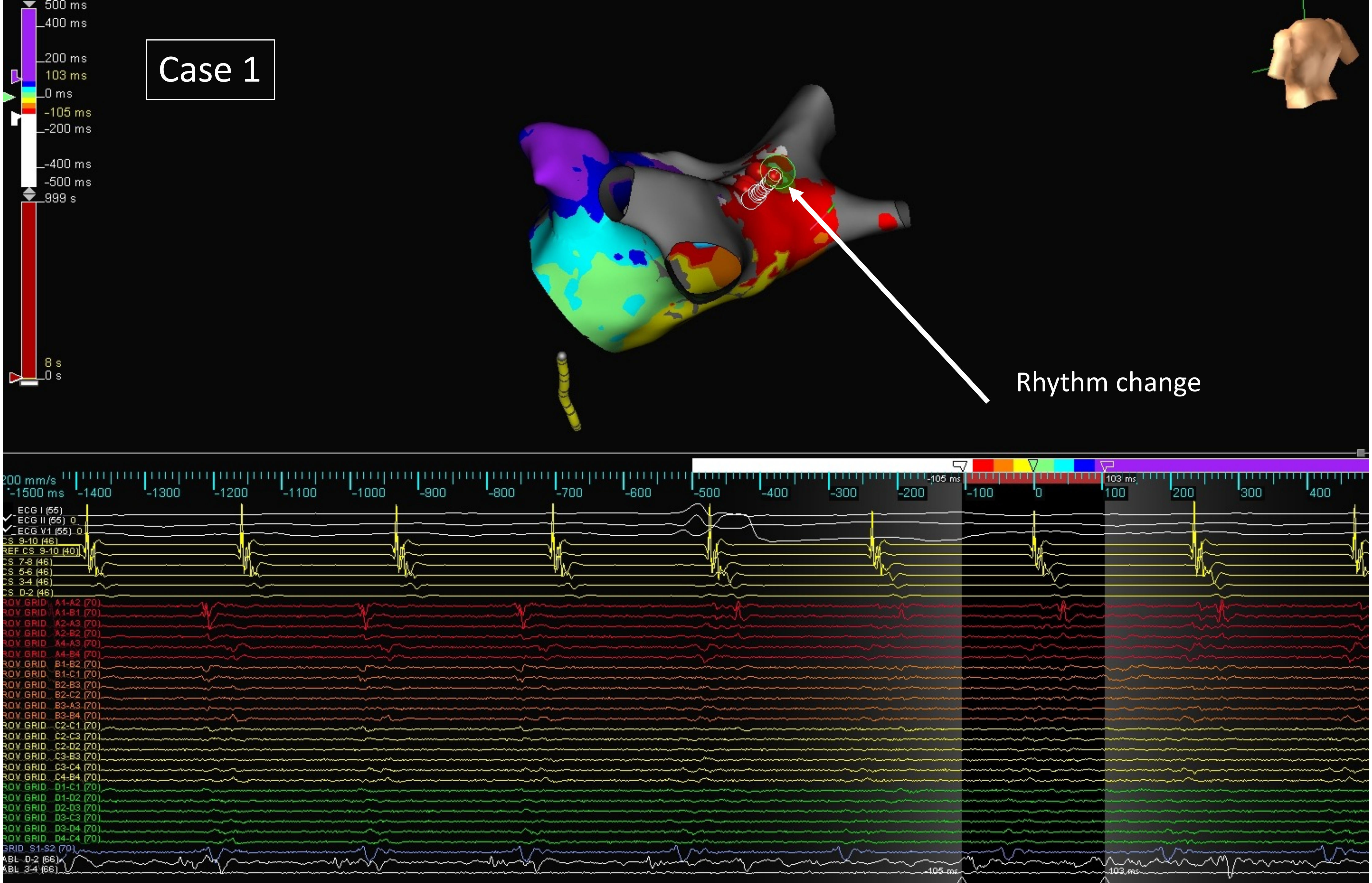
Case 1
Rhythm change
500 ms
400 ms
200 ms
103 ms
0 ms
-105 ms
-200 ms
-400 ms
-500 ms
999 s
8 s
0 s
200 mm/s
-1500 ms -1400 -1300 -1200 -1100 -1000 -900 -800 -700 -600 -500 -400 -300 -200 -100 0 100 200 300 400
ECG I (55)
ECG II (55)
ECG V1 (55)
CS 9-10 (46)
REF CS 9-10 (40)
CS 7-8 (46)
CS 5-6 (46)
CS 3-4 (46)
CS D-2 (46)
ROV GRID A1-A2 (70)
ROV GRID A1-B1 (70)
ROV GRID A2-A3 (70)
ROV GRID A2-B2 (70)
ROV GRID A4-A3 (70)
ROV GRID A4-B4 (70)
ROV GRID B1-B2 (70)
ROV GRID B1-C1 (70)
ROV GRID B2-B3 (70)
ROV GRID B2-C2 (70)
ROV GRID B3-A3 (70)
ROV GRID B3-B4 (70)
ROV GRID C2-C1 (70)
ROV GRID C2-C3 (70)
ROV GRID C2-D2 (70)
ROV GRID C3-B3 (70)
ROV GRID C3-C4 (70)
ROV GRID C4-B4 (70)
ROV GRID D1-C1 (70)
ROV GRID D1-D2 (70)
ROV GRID D2-D3 (70)
ROV GRID D3-C3 (70)
ROV GRID D3-D4 (70)
ROV GRID D4-C4 (70)
GRID S1-S2 (70)
ABL D-2 (66)
ABL 3-4 (66)

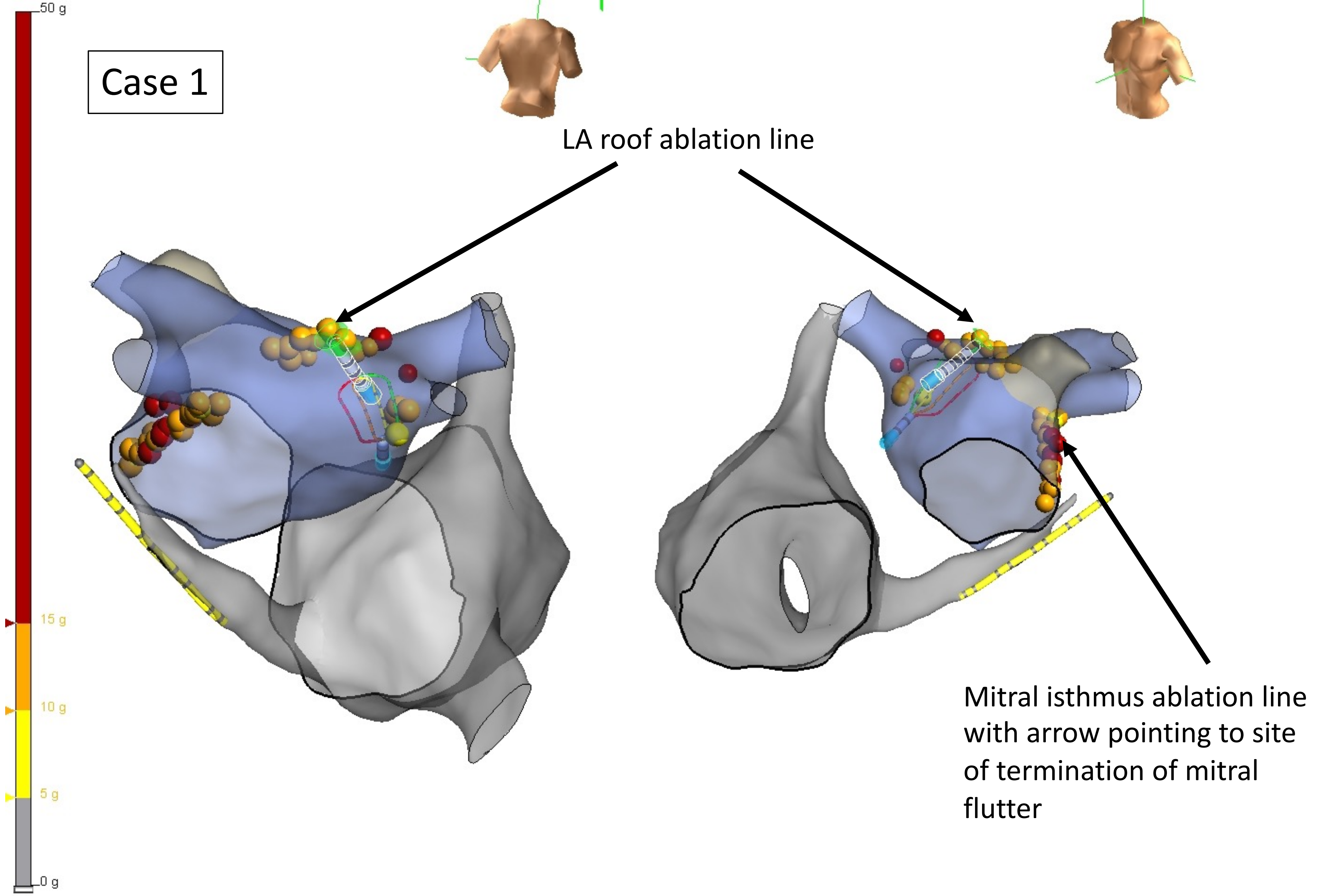
50 g
15 g
10 g
5 g
0 g
Case 1
LA roof ablation line
Mitral isthmus ablation line with arrow pointing to site of termination of mitral flutter

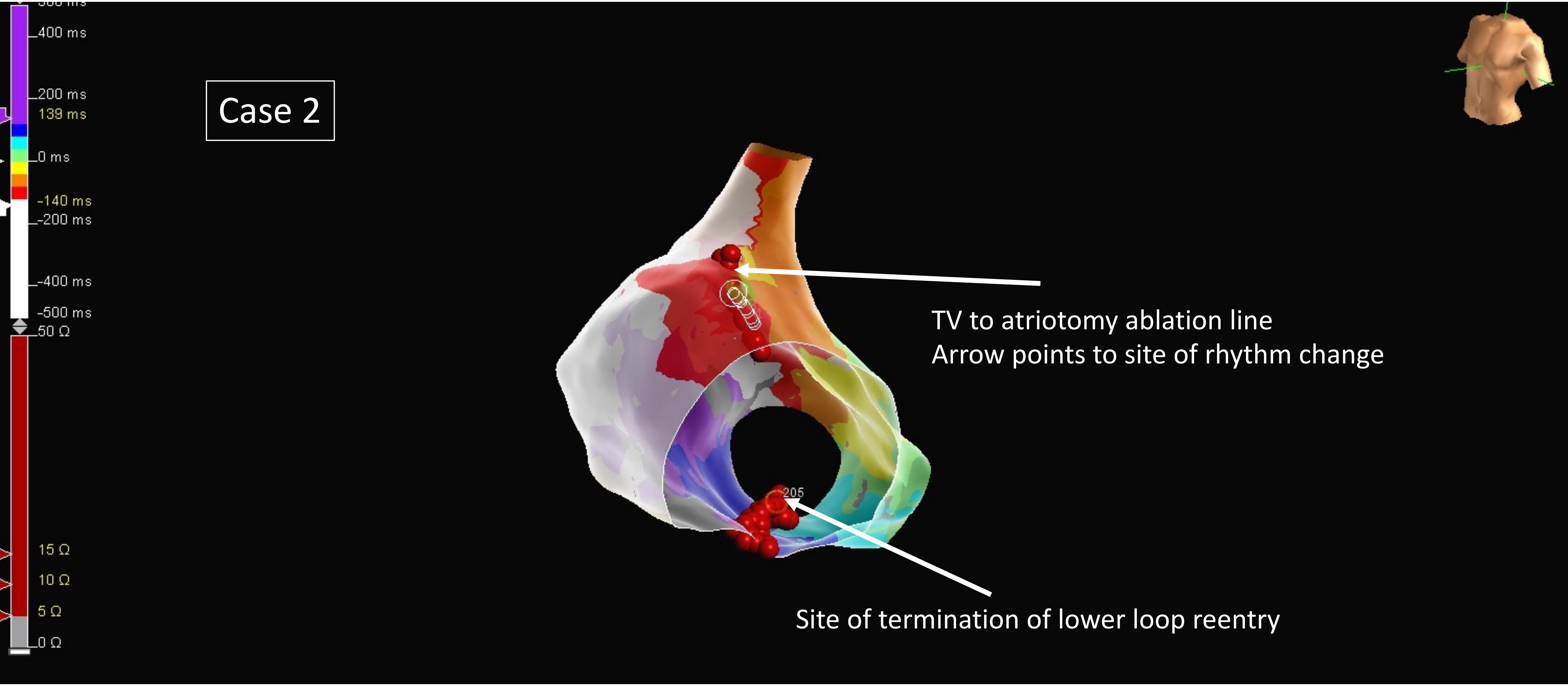
400 ms
200 ms
139 ms
0 ms
-140 ms
-200 ms
-400 ms
-500 ms
50 Ω
15 Ω
10 Ω
5 Ω
0 Ω
Case 2
205
TV to atriotomy ablation line
Arrow points to site of rhythm change
Site of termination of lower loop reentry

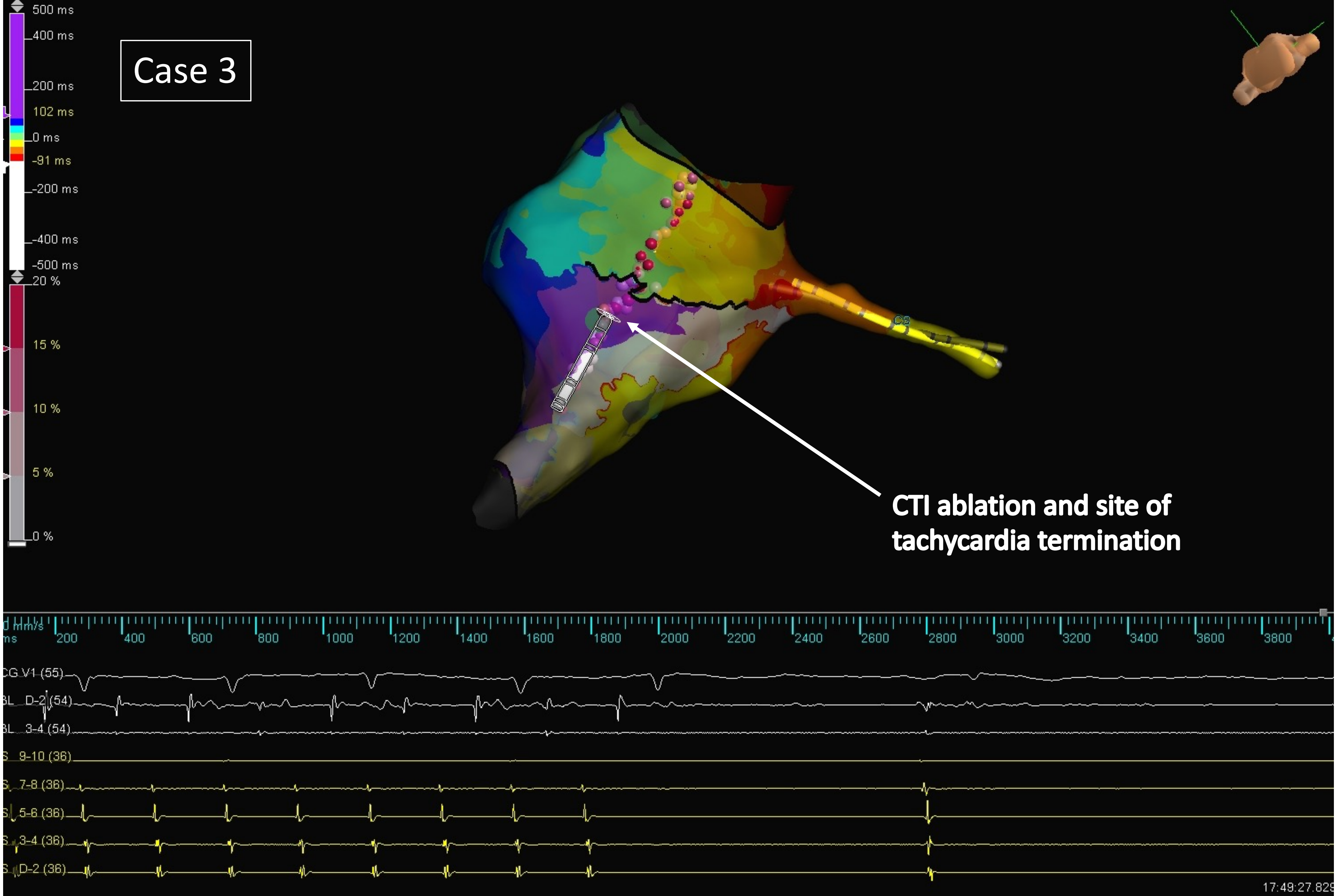
Case 3
CTI ablation and site of tachycardia termination
500 ms
400 ms
200 ms
102 ms
0 ms
-91 ms
-200 ms
-400 ms
-500 ms
20 %
15 %
10 %
5 %
0 %
CS
ms
200
400
600
800
1000
1200
1400
1600
1800
2000
2200
2400
2600
2800
3000
3200
3400
3600
3800
CG V1 (55)
BL D-2 (54)
BL 3-4 (54)
S 9-10 (36)
S 7-8 (36)
S 5-6 (36)
S 3-4 (36)
S D-2 (36)
17:49:27.829
