# Supplementary material for: Rapid detection of isthmus block and rhythm change using local electrogram changes during complex atrial flutter ablation
Source: Europace. 2022 Sep 15;25(2):756–61. doi: 10.1093/europace/euac161 (PMC10103578; doi:10.1093/europace/euac161)
Supplement: euac161_Supplementary_Data [file euac161_supplementary_data.zip › supplementalFile.docx]

**Procedural details for case 1**

Patient underwent trans-septal puncture under intracardiac echocardiogram and continuous pressure monitoring. Following trans-septal puncture, a Toray^TM^ looped wire was advanced into the left atrium. Ablation catheter (Tacticath^TM^, Abbott Lab) was advanced into the left atrium alongside the Toray wire. The trans-septal sheath is advanced over the Toray wire and a HD grid^TM^ mapping catheter is advanced into the LA via the sheath. A decapolar deflectable catheter placed in the coronary sinus serves as the reference electrode for activation mapping. After the presenting rhythm was determined to be a dual loop reentry around mitral valve and left pulmonary veins, we placed the HD grid^TM^ catheter on the Rwf side of the roof ablation line. Roof line was started near the left superior pulmonary vein and continued towards the right superior pulmonary vein posteriorly. Change in activation sequence on the HD grid indicating roof block occurred with completion of 3/4^th^ of the ablation line. Anatomic completion of the line was done up to the line of block around the right superior pulmonary vein.

**Case 1 Videos**

**Video 1:** **The left panel** shows propagation map of dual loop reentry around the mitral valve and left pulmonary veins; the common isthmus is the mitral isthmus between left inferior pulmonary vein and mitral valve. The non-common isthmus is the left atrial roof.

**The right panel** shows propagation map after completion of roof line. Counterclockwise mitral flutter continues, and activation of the Awf area remains unchanged while the Rwf activation is reversed.

**Video 2** demonstrates the instantaneous display of activation sequence change on the HD grid catheter using LiveView^TM^ technology. The activation change is color coded and correlates to electrogram changes in Figure 3C

**Case 2 Videos**

**Video 3** shows propagation of two independent simultaneous reentry in the right atrium- one seen around atriotomy scar in front of the superior vena cava and a second one seen around the inferior vena cava. There is no common isthmus.

**Video 4** shows right atrial propagation map after creation of tricuspid valve to atriotomy scar ablation line. There is no further reentry around the atriotomy, but continued reentry is seen around the inferior vena cava. Note collision of activation at the ablation line between tricuspid valve and atriotomy scar.

**Case 3 Video**

**Video 5** shows simultaneous intra cavotricuspid isthmus and tricuspid valve reentry forming a dual loop reentry
